# A Highly Sensitive Electrochemical Immunosensor for Cortisol Detection

**DOI:** 10.3390/bios15050321

**Published:** 2025-05-17

**Authors:** Pritu Parna Sarkar, Ali Ashraf, Ahmed Hasnain Jalal, Fahmida Alam, Nazmul Islam

**Affiliations:** 1 Department of Mechanical Engineering, The University of Texas Rio Grande Valley, Edinburg, TX 78539, USA; prituparna.sarkar01@utrgv.edu; 2 Department of Mechanical Engineering, The University of South Florida, Tampa, FL 33620, USA; aliashraf@usf.edu; 3 Department of Electrical & Computer Engineering, The University of Texas Rio Grande Valley, Edinburg, TX 78539, USA; ahmed.jalal@utrgv.edu (A.H.J.); fahmida.alam@utrgv.edu (F.A.)

**Keywords:** printable sensor design, cortisol, antibody-based immunosensor, flexible sensor applications, sensor coatings

## Abstract

In this research, an interdigitated gear-shaped working electrode is presented for cortisol sensing. Overall, the sensor was designed in a three-electrode system and was fabricated using direct laser scribing. A synthesized conductive ink based on graphene and polyaniline was further employed to enhance the electrochemical performance of the sensor. Scanning electron microscopy (SEM) and Fourier transform infrared (FTIR) spectroscopy were employed for physicochemical characterization of the laser-induced graphene (LIG) sensor. Cortisol, a biomarker essential in detecting stress, was detected both in phosphate-buffered saline (PBS, pH = 7.4) and human serum within a linear range of 100 ng/mL to 100 µg/mL. Ferri/ferrocyanide was employed as the redox probe to detect cortisol in PBS. The electrochemical performance of the developed sensor was assessed via differential pulse voltammetry (DPV), cyclic voltammetry (CV), electrochemical impedance spectroscopy (EIS), and chronoamperometry. The electrochemical performance demonstrates high sensitivity and selectivity alongside strong repeatability (relative standard deviation (RSD) = 3.8%, n = 4) and reproducibility (RSD = 5.85%, n = 5). Overall, these results highlight the sensor’s reliability, high sensitivity, and repeatability and reproducibility in the detection of cortisol. The sensor successfully detected cortisol in the complex medium of human serum and effectively distinguished it in a ternary mixture containing cortisol and dopamine. Also, the use of direct laser writing on Kapton film makes the approach cost-effective and thus disposable, making it suitable for chronic stress diagnostics and neurological research applications.

## 1. Introduction

Stress is defined as a feeling caused by emotional tension, and it can occur in situations in which one must respond to demands or pressures beyond the capability of the individual [1]. According to the National Library of Medicine, a certain amount of stress is essential to enhance performance and productivity. However, when stress is not managed, it poses a threat to the happiness and health of modern-day people [2]. It also plays a crucial role in various mental disorders, such as anxiety and seizures [3]. More than 75% of adults report experiencing stress symptoms, including headaches, fatigue, and depression. Among U.S. workers, 83% say they deal with stress related to their jobs. Nearly half of all U.S. adults (49%) acknowledge that stress has negatively impacted their behavior [4,5]. In total, 53% of adults say that stress has a great impact on their mental health. This increasing level of stress is consequently affecting mental health and making people more anxious. In 2024, 43% of adults reported feeling more anxious than they had felt in the previous year, an increase from 37% in 2023 and 32% in 2022 [6]. Consequently, the continuous monitoring of stress levels is becoming increasingly important, particularly for those who work under immense pressure, such as armed forces personnel, police officers, and firefighters. When an individual is stressed, the body releases specific biomarkers into bodily fluids (such as blood, urine, saliva, and sweat) the concentration of which fluctuates with the level of stress of the individual. The most common stress biomarkers in bodily fluids include cortisol, interleukin-6 (IL-6), serotonin, oxytocin, cardiac troponin, epinephrine, and tumor necrosis factor-alpha (TNF-α) [7]. Monitoring these biomarkers can provide insights into whether a person is experiencing stress.

Among these biomarkers, cortisol is widely regarded as one of the most essential and relevant biomarkers for the assessment and monitoring of both psychological and physiological stress [8]. It is commonly referred to as the “stress hormone” due to its association with negative health outcomes and psychological stress [7,9]. Cortisol is a small-molecule steroid hormone secreted by the adrenal glands located above each kidney. The concentration of cortisol varies across different bodily fluids and according to different times of day. Abnormal cortisol levels can lead to issues such as low blood glucose, obesity, depression, and diabetes. Consequently, measuring cortisol levels efficiently and accurately is crucial [10]. Due to the low concentrations of cortisol and its small molecular size, a highly sensitive sensor is necessary for detection [11].

Current approaches used to detect cortisol in various bodily fluids have evolved from conventional laboratory-based techniques, including liquid chromatography–tandem mass spectrometry (LC-MS/MS) [10] and enzyme-linked immunosorbent assays (ELISA) [12]. Additional analytical techniques, including capillary electrophoresis-based immunosorbent assays (CE-IA) [13], surface plasmon resonance (SPR) [14,15], high-performance liquid chromatography (HPLC) [16,17], and electro-chemiluminescent immunoassay (ECLIA) [18,19], provide effective cortisol measurement. Although these methodologies facilitate the identification of cortisol at sub-ng/mL concentrations with remarkable precision and exhibit unparalleled sensitivity and selectivity, they are not devoid of intrinsic limitations. Despite their analytical accuracy, these methods often necessitate complex sample preparation, large sample volumes, and skilled operators. Furthermore, the possibilities of false-positive results and prolonged assay durations further hinder their practical implementation in field applications [8,11,13].

To address these challenges, electrochemical sensors have gained prominence as a solution, offering a label-free detection approach. This approach relies on detecting changes in the electrical properties of a conductive substrate when an analyte interacts with a surface functionalized with specific antibodies, molecularly imprinted polymers (MIPs), or aptamers [20,21,22]. The fabrication of electrochemical biosensors predominantly involves screen-printed electrodes (SPEs), a technique that has gained popularity since Tour and colleagues [23] first introduced the fabrication of porous graphene films from polymeric substrates via laser irradiation. Among the most common methods, polyimide tapes and films are employed for photothermal conversion, enabling the transformation of polymers into the 3D, porous graphene structures used in electrode manufacturing [24,25,26].

In this research, we developed a cost-effective, disposable graphene-based interdigitated electrochemical immunosensor with a unique gear-shaped design capable of rapid cortisol detection. The sensor was fabricated using a cost-effective direct laser-writing technique on Kapton film. We performed the surface characterization employing Fourier transform infrared spectroscopy (FTIR) as well as scanning electron microscopy (SEM), confirming the successful formation of the graphene on Kapton as well as the polyaniline layer on top of that. To enhance antibody immobilization, the sensor surface was treated with brief UV–ozone treatment to increase the number of active sites; this was followed by functionalization using N-(3-Dimethylaminopropyl)-N’-Ethylcarbodiimide Hydrochloride/N-Hydroxysuccinimide (EDC/NHS) chemistry. Our initial experiments focused on assessing the sensor’s cortisol detection capabilities using cyclic voltammetry (CV), differential pulse voltammetry (DPV), chronoamperometry, and electrochemical impedance spectroscopy (EIS) in the μg/mL range. The sensor also demonstrated strong repeatability, reproducibility, and selectivity, as well as effective performance in complex media like human serum.

## 2. Materials and Instruments

### 2.1. Chemical Materials and Instruments

Cortisol solution (CAS No: 50-23-7, concentration 1.0 mg/mL in methanol), aniline (CAS number: 62-53-3), phosphate-buffered solution (10 M, a pH of 7.4), phytic acid (CAS No: 83-86-3), silver ink, and human serum (H3667) were obtained from Sigma Aldrich, St. Louis, MO, USA. Anti-cortisol antibody (XM210) was acquired from Abcam (Boston, MA, USA). The antibody was supplied at a concentration of 2 mg/mL and was suspended in phosphate-buffered saline (PBS, pH 7.4) containing 0.09% sodium azide as a preservative. Polydimethylsiloxane (PDMS) was synthesized by combining SYLGARD™ 184 Silicone Elastomer Base with SYLGARD™ 184 Silicone Elastomer Curing Agent in a 10:1 ratio. Graphene nanoflakes (CAS No: 7782-42-5) (specific surface area: 750 m^2^/g, conductivity: 8000 S/m), N-Hydroxysuccinimide (NHS) (CAS No: 6066-82-6), N-(3-Dimethylaminopropyl)-N’-Ethylcarbodiimide Hydrochloride (EDC) (CAS No: 25952-53-8), potassium hexacyanoferrate (III) (CAS No: 13746-66-2), and potassium hexacyanoferrate (II) trihydrate (CAS No: 14459-95-1) were also procured from Sigma Aldrich, St. Louis, MO, USA. Kapton polyimide film (type: HN; thickness of 0.152 mm) were purchased from McMaster-Carr (Atlanta, GA, USA). Sodium dodecyl sulfate (CAS 151-21-3) was purchased from fisher bioreagents (Waltham, MA, USA).

The three-electrode gear-shaped system was designed using the vector graphics software Autodesk Inventor (2024, version 24.2), and the laser-induced graphene (LIG) sensors were fabricated with a Glowforge Pro (Glowforge, Seattle, WA, USA). For material characterization, we employed a field emission scanning electron microscope from Zeiss (Zeiss, Oberkochen, Germany); a Bruker Vertex 70 Fourier Transform Infrared Spectrometer (FTIR) (Billerica, MA, USA) was used for FTIR analysis. A Novascan digital UV ozone system—OES-1000D-Ozone Elimination System (Novascan, Ames, IA, USA) was used for the ozonolysis treatment. We used a DRP-CAC4MMH cable to connect the three-electrode sensor to the Autolab Potentiostat PGSTAT302N (Metrohm, Riverview, FL, USA), and conducted all electrochemical experiments. The software associated with the potentiostat is Nova 2.1. The data obtained was further processed, and all graphs were obtained, using Origin Pro (Origin Lab Corporation, Northampton, MA, USA).

### 2.2. G-PANI Ink Synthesis and Fabrication of Anti-Cortisol Antibody-Based Immunosensors

**Figure 1:**
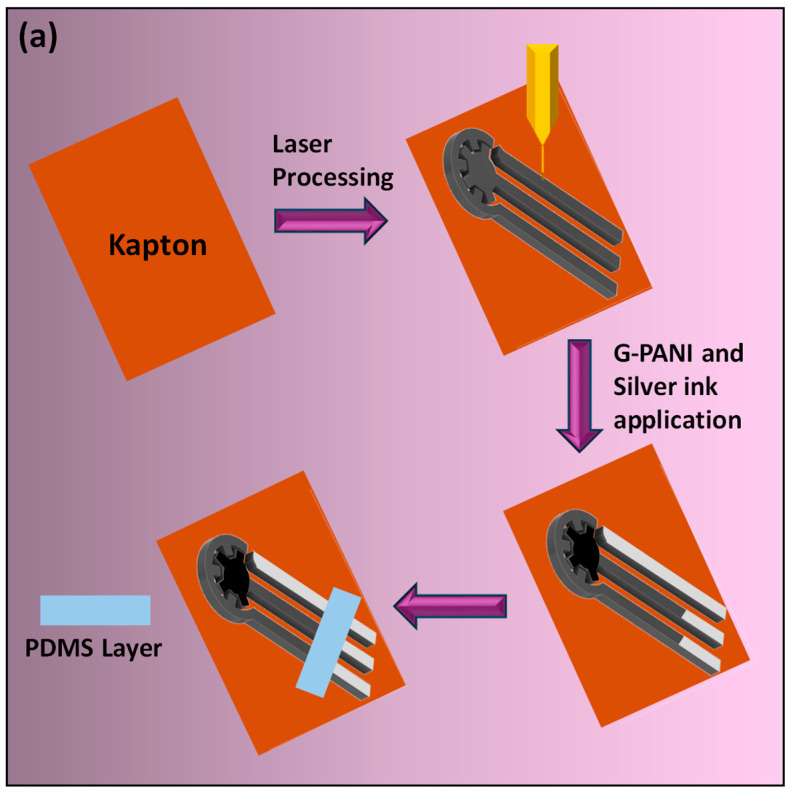

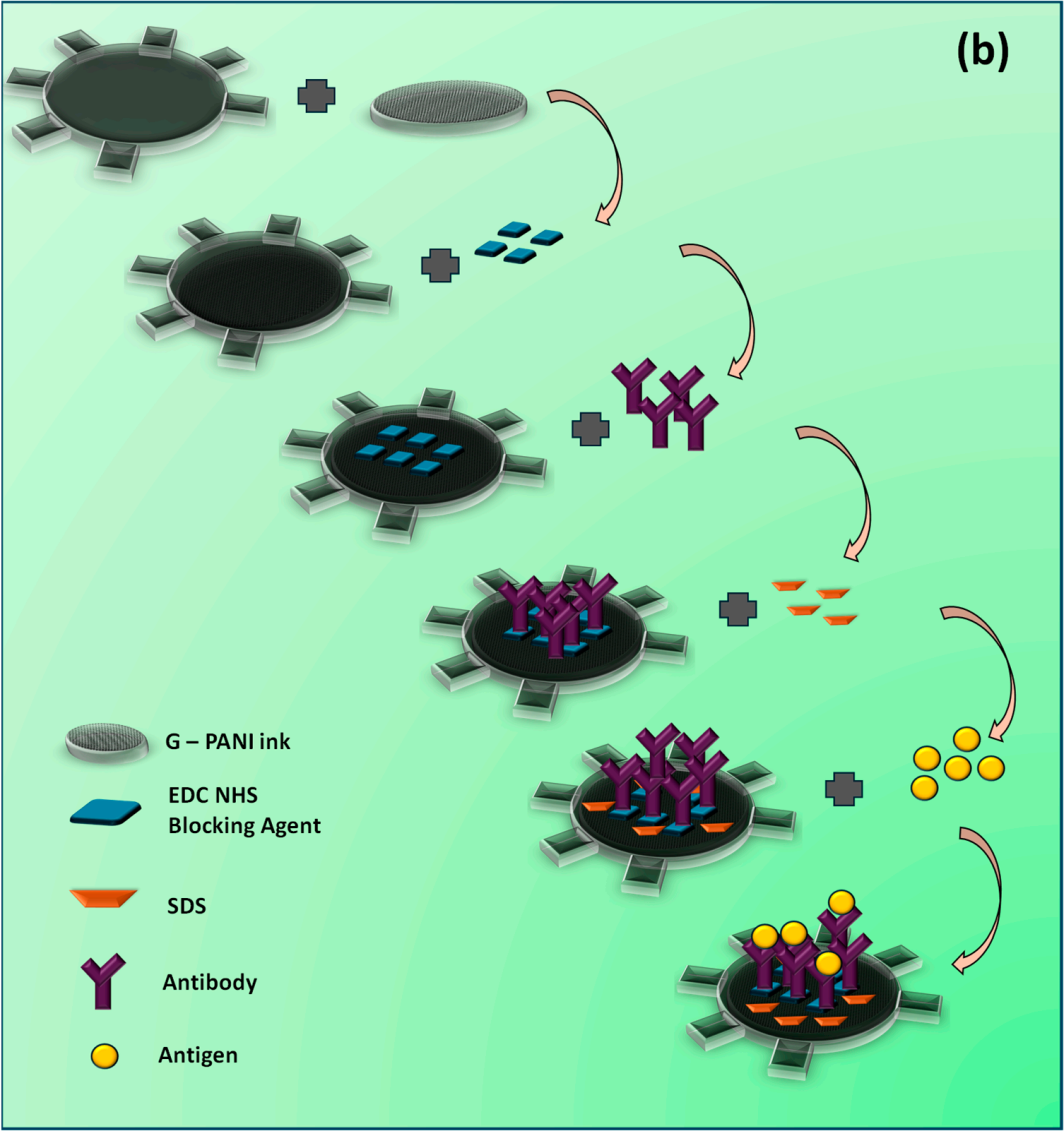
Schematic illustration of (**a**) the fabrication process of the LIG sensor, including laser engraving on Kapton film followed by sequential addition of G-PANI ink, silver ink, and a PDMS layer, and (**b**) the immobilization of cortisol antibody on the working electrode.

The synthesis of graphene–polyaniline (G-PANI) ink and electrode fabrication followed the methods described in our previous work [27,28]. Briefly, 2 mL of polyaniline, 4 mL of phytic acid, 6 mL of deionized water, and 1 g of graphene nanoflakes were mixed using a planetary mixer for the G-PANI ink. Polyaniline (PANI) was synthesized in situ through the polymerization of aniline in the presence of phytic acid, which served both as a dopant and as a component of the reaction medium. The novel interdigitated gear-shaped sensor was fabricated via direct laser printing on Kapton film to produce laser-induced graphene (LIG); this was followed by modification of the working electrode with G-PANI ink, and the modification of the contact pad and reference electrode with silver ink. The fabricated LIG was used as counter electrode (CE), and provided good electrical conductivity and a sufficiently large electroactive area to support the current levels between the working electrode and counter electrode. The reference electrode (RE) was prepared by manually applying a layer of commercially available silver ink onto the LIG substrate, followed by air drying. The three-electrode system was chosen for its preciseness in quantification. The detailed reason for selecting the gear-shaped design and the method for calculating the effective surface area are discussed in detail in our previous work [29]. In short, we performed CV on an interdigitated gear-shaped sensor on Kapton film, as well as on a commercially available sensor and a sensor on Kapton film without the modified design. We then calculated the effective surface area of the corresponding CV graphs using the quasi-Randles–Sevcik equation; this was found to be 3.70 cm^2^ for the improved design. This modified WE design significantly enhances the active surface area by 94.52%, when compared to Metrohm DropSens 110 screen-printed sensors, and when compared to printed circular sensors, the area increases by 57%, as calculated in our previous paper [30]. A PDMS layer was manually applied between the working zone and contact pads using Q-tips, so the analyte would not spread to the conductive contact-pad region (shown in Figure 1a). The sensor was left to dry overnight at ~25 °C.

### 2.3. Immobilization of Antibody upon the Working Electrode’s Surface

To immobilize the cortisol antibody on the working electrode, the sensor first underwent ozonolysis at 40 °C for 3.5 min to introduce carboxyl groups (−C(=O)−OH) on the surface. After that, 10 µL of 0.4 M EDC was applied to activate the carboxyl group, and the sensor was kept in a dark environment for 2 h; this was followed by the addition of 10 µL of 0.4 M NHS solution and another 2 h incubation in the dark. Next, 10 µL of cortisol antibody was added and left for 1 h to conjugate with NHS molecules. After the immobilization, the 10% (*w*/*v*) SDS solution was used as a surfactant to modulate surface interactions and minimize nonspecific adsorption and left for another 1 h. After that, the sensor was ready for detection. If it is not used immediately, it can be preserved at 4 °C until it is used. The whole procedure is shown in Figure 1b.

### 2.4. Electrochemical Measurement of Cortisol

A DRP-CAC4MMH cable was used to connect the three-electrode sensor to the Autolab Potentiostat for electrochemical analysis. CV, DPV, chronoamperometry, and EIS were employed to evaluate the performance of the sensor. For CV measurements, the parameters were set to a potential range of −0.5 V to 0.6 V with a scan rate of 50 mV/s. EIS was conducted with a start frequency of 10^6^ Hz, a stop frequency of 1 Hz, 10 frequencies per decade, a DC voltage of 0.01 V, and an AC voltage of 0.01 V sinusoidal. Chronoamperometric measurements of cortisol at the LIG sensor were conducted with the working electrode (WE) potential set at 0.3 V for both the initial and subsequent potential steps. For DPV analysis, a scan range of 0–0.7 V, step 0.01 V and modulation amplitude 0.025 V were used. In all experiments, 10 µL of each analyte concentration was applied to the working electrode (WE); this was followed by a 10 min incubation. Afterward, an additional 10 µL of the same concentration, along with 10 µL of ferri/ferrocyanide, were added before running the experiment. Here, the ferri/ferrocyanide solution comprises redox probes, which is crucial in an electrochemical sensor, because a redox probe acts as a “messenger” molecule, facilitating the transfer of electrons between the electrode’s surface and the analyte of interest, allowing for the detection and quantification of the analyte by measuring the resulting electrical signal generated during the redox reaction; essentially, it enables the sensor to accurately measure the redox potential of a sample by providing a reference point for electron exchange [31,32]. Between tests, the sensor was thoroughly rinsed with 1× PBS and deionized (DI) water.

## 3. Results and Discussion

### 3.1. Characterization of the Prepared LIG/G-PANI Electrode

**Figure 2:**
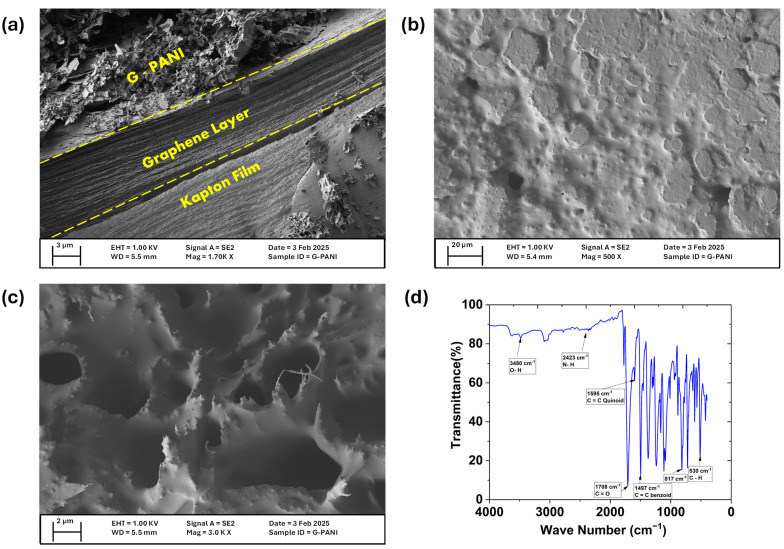
(**a**) Cross-sectional view of the G-PANI ink-coated LIG sensor showing three distinct layers of Kapton, Graphene and G-PANI ink. (**b**) SEM image of a thin and evenly distributed layer of PANI on the graphene. (**c**) The porous top view of the G-PANI ink, which significantly increases the surface area. (**d**) Fourier transform infrared spectroscopy (FTIR) of the LIG/G-PANI sensor, showing the peaks from both PANI and graphene.

The cross-sectional side view and top view of the SEM image clearly reveal three distinct layers: the intact Kapton film, the laser-induced graphene layer, and the porous PANI layer (respectively shown in Figure 2a,b). A layer of PANI on the graphene indicates successful polymerization and strong interaction between the two materials. The presence of some folded edges is attributed to the 2D nature of graphene. The homogeneous texture suggests efficient and even polymerization of PANI onto the graphene. When zoomed to 3000X (Figure 2c), we observe some distinct rough and porous structures which are beneficial for our electrochemical applications as they significantly increase surface area and conductivity. Due to the interdigitated gear-shape design of the working electrode, the surface area is already greater than the commercially available one, while the additional PANI layer enhances electrochemical performance and further contributes to this by making the surface area more prominent. A larger surface area enhances the absorption of biomolecules, improving detection sensitivity, and leading to more accurate cortisol detection [33,34].

The FTIR spectrum of pure PANI shows prominent peaks at 3480 cm^−1^, 2420 cm^−1^, ~1470–1500 cm^−1^, and 540 cm^−1^. Key peaks include the points at 3480 cm^−1^, indicating O–H stretching; 2420 cm^−1^ represents asymmetric N–H stretching; the range of ~1470–1500 cm^−1^ indicates the C=C stretching in benzenoid rings; and the peak at 540 cm^−1^ corresponds to the C–H out-of-plane bending mode. A peak around ~800–830 cm^−1^ confirms the out-of-plane C–H bending, verifying the PANI ring structure. The peak at ~1720 cm^−1^ is related to C=O stretching, which can be attributed to carboxylic or carbonyl groups in graphene oxide (GO). The FTIR spectra of the synthesized graphene–polyaniline nanocomposites retain the prominent peaks of both PANI and graphene (demonstrated in Figure 2d), with slight changes in intensity, indicating the successful incorporation of PANI onto the graphene surface [34]. Also, in our previous work on a similar LIG sensor, we have demonstrated the graphene layer through XPS analysis [28].

### 3.2. Cyclic Voltammetry

**Figure 3:**
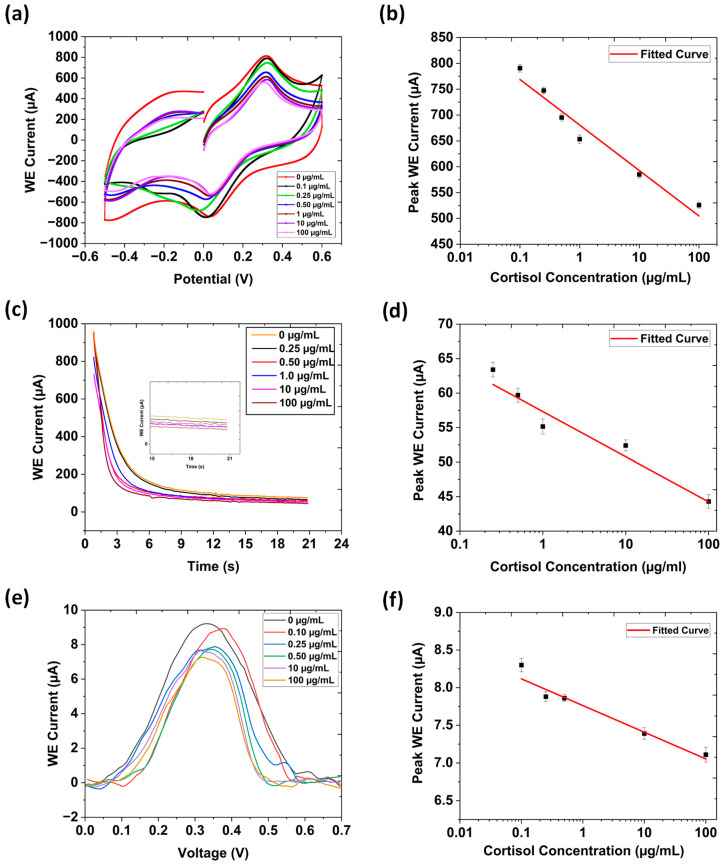
(**a**) CV responses obtained for interdigitated gear-shaped LIG/G-PANI sensor as a function of different cortisol concentrations (100 ng/mL to 100 μg/mL) in 1X PBS (pH = 7.4). (**b**) Linear plot of the oxidation peak currents vs. different cortisol concentrations. (**c**) Chronoamperometry response obtained for the sensor as a function of different cortisol concentrations (250 ng/mL to 100 μg/mL) in 1X PBS (pH = 7.4) (inset: an enlarged version of the steady-state current from 12 s to 20 s). (**d**) Linear plot of the stable currents vs. different cortisol concentrations. (**e**) DPV response obtained for the sensor as a function of different cortisol concentrations (100 ng/mL to 100 μg/mL) in 1X PBS (pH = 7.4). (**f**) Corresponding linear plot of the peak WE currents vs. different cortisol concentrations.

The electrochemical response of the interdigitated immune electrode was evaluated using CV in a three-electrode system with phosphate-buffered saline (PBS, pH 7.4), across cortisol concentrations ranging from 100 ng/mL to 100 μg/mL; this is presented in Figure 3a. A progressive decline in the electrochemical current response was observed with increasing cortisol concentrations due to the formation of an insulating immune complex between the cortisol and its antibody. As more cortisol molecules became bound during the immunological binding process, electron transport slowed. This hindrance increased interfacial impedance, while decreasing charge-transfer kinetics. As a result, the electron transfer rate slowed, and the maximum current decreased in value. At higher cortisol concentrations, this impediment is further exacerbated. Additionally, immunocomplex formation reduces the active electrode area available for redox reactions, contributing to the decline in current response [35].

The calibration curve depicting the relationship between the current response and the logarithm of cortisol concentration is depicted in Figure 3b. The derived linear regression equation, ΔI (μA) = −85.715 log [cortisol conc. (μg/mL)] + 683.843, exhibited a strong correlation coefficient (R^2^ = 0.966), indicating excellent linearity. The limit of detection (LOD) calculated from the CV calibration curve was 0.0813 μg/mL, using the formula LOD = σ/0.13 × S, where (σ stands for the standard deviation of the response and S is the slope). Error bars indicate the results of four consecutive experiments (n = 4).

### 3.3. Chronoamperometry

This study conducted chronoamperometric measurements by applying a potential of 0.3 V to the working electrode, with the corresponding current being recorded over 60 s (Figure 3b). The obtained chronoamperometric profile demonstrated a clear exposure–response relationship with significant current variations upon cortisol binding. The formation of the cortisol antibody–cortisol complex governed the sensor’s response. The applied open circuit potential (0.3 V vs. Ag/AgCl) facilitated the faradaic reduction, leading to an initial rapid decrease in current followed by a steady-state plateau. Upon cortisol binding, a notable decrease in current response was observed, which is characteristic of a “signal-off” affinity-based sensing mechanism. The linear regression equation was determined as I = −6.540 log [cortisol conc. (μg/mL)] + 57.320, with a correlation coefficient (R^2^ = 0.959), and the LOD was 0.105 μg/mL, as indicated in Figure 3d. These results demonstrate the biosensor’s capability for real-time cortisol detection with high sensitivity and reliability.

### 3.4. Differential Pulse Voltammetry

Differential pulse voltammetry (DPV) was employed to evaluate the sensing characteristics of the immunoelectrode towards cortisol detection, using an applied potential range of 0.0 V to 0.7 V. The DPV graph was plotted after baseline correction (Figure 3e). As cortisol concentration increased, the DPV peak current decreased due to the accumulation of cortisol molecules on the electrode’s surface. A calibration curve was constructed by plotting the DPV peak current and the cortisol concentration in Figure 3f, resulting in a regression equation of ΔI = −0.351 log [cortisol conc. (μg/mL)] + 7.795 with a correlation coefficient of (R^2^ = 0.925). These results confirm that the immunosensor demonstrated excellent sensitivity and potential for precise cortisol detection.

### 3.5. Electrochemical Impedance Spectroscopy (EIS)

Electrochemical impedance spectroscopy (EIS) was employed to assess the interfacial properties of the antibody-functionalized biosensor for cortisol detection. Nyquist plots, depicting the relationship between imaginary and real impedance, were analyzed to determine charge-transfer resistance (R_ct_), a critical parameter in biosensing performance. The high-frequency region corresponds to charge-transfer kinetics, while the low-frequency region is associated with diffusion-limited processes. The semicircle diameter in the Nyquist plot represents R_ct_, which is directly influenced by the analyte concentration at the electrode’s surface.

**Figure 4:**
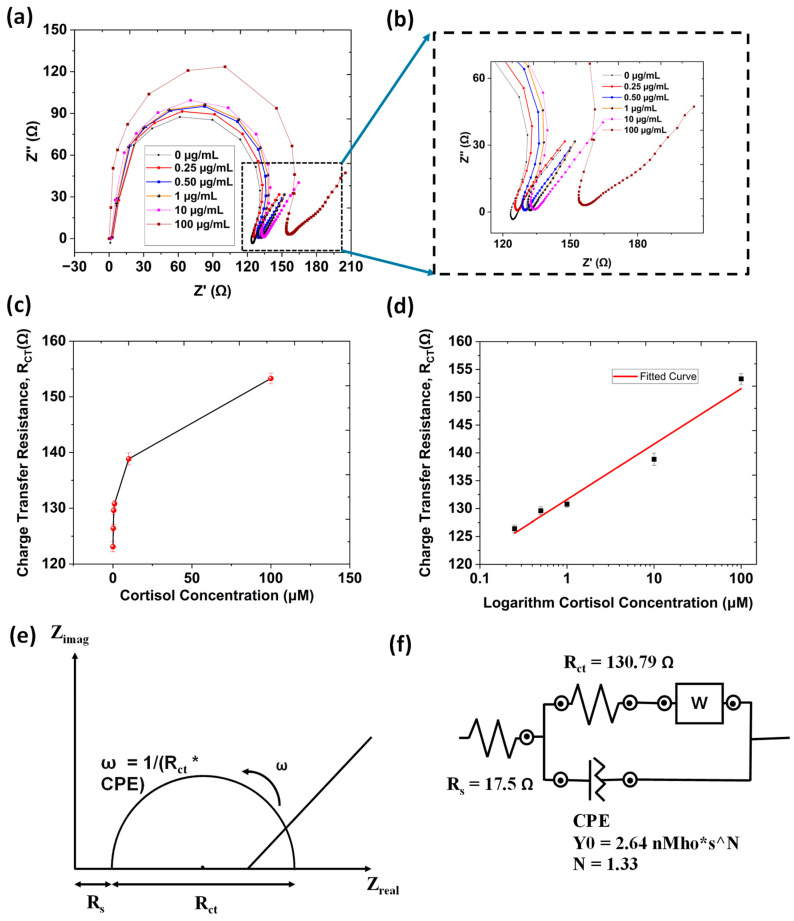
(**a**) EIS characterization of cortisol detection from 0.25 µg/mL to 100 µg/mL in 1× PBS (pH = 7.4). (**b**) Enlarged view of the previous EIS characterization. (**c**) Charge-transfer resistance plot with different cortisol concentrations. (**d**) Fitted linear plot between charge-transfer resistance and logarithm of cortisol concentration for determining the limit of detection for the cortisol. (**e**) Generalized Nyquist plot. (**f**) Randles equivalent circuit for the cortisol concentration of 1 μg/mL.

The EIS analysis of the gear-shaped interdigitated biosensor for different concentrations of cortisol is shown in Figure 4a,b. The baseline signal was established using a blank buffer solution. A progressive increase in the charge-transfer resistance (R_ct_) was observed as cortisol concentrations increased from 0.10 to 100 µg/mL (Figure 4c), indicating the formation of insulating immune complexes that hinder electron transfer. The rising resistance values justify the declining current values observed in CV, DPV, and chronoamperometry with the increase in cortisol concentrations. The calibration curve of ΔR_ct_ versus the logarithm of cortisol concentration (shown in Figure 4d) exhibited strong linearity (R^2^ = 0.976), with a regression equation of ΔR_ct_ = 9.94 log [C] + 131.60 and LOD of 0.0577 μg/mL. Figure 4e represents a general Nyquist plot, which is a graphical representation of the impedance data, showing the real part of the impedance (Z′) on the *x*-axis and the imaginary part (Z″) on the *y*-axis, which is similar to the plot that we have shown in Figure 4a. The semicircle of the plot represents the charge-transfer resistance and double layer capacitance, while a straight line at lower frequencies represents Warburg diffusion [36]. A Randles equivalent circuit was used to further fit the Nyquist plot obtained in Figure 4a,b for all concentrations. In Figure 4f a Randles equivalent circuit for the cortisol concentration of 1 μg/mL is shown. The other values of the components are shown in Table 1. These EIS results support the earlier findings from CV, DPV, and chronoamperometry, confirming the efficacy of the developed biosensor in detecting cortisol with high sensitivity, reproducibility, and reliability, making the biosensor a promising candidate for real-time, label-free, and cost-effective cortisol monitoring.

The following, Table 2, provides a summary of the performance analyses for several electrochemical sensors used for cortisol detection, including the one used in this work.

**Table 2:** Comparison of cortisol detection performance for different biosensors.

Electrode	Technique	Linear Range	Limit of Detection (LOD)	Reference
IrO_x_ modified reference electrode/Au	EIS	1 ng/mL to 1 mg/mL	11.85 pg/mL	[37]
Inject-printed Au	EIS	5–20 ng/mL	1.18 ng/mL	[38]
Au single-atom nanozymes	Square wave voltammetry (SWV)	0.15–300 (ng mL^−1^)	0.48 pg mL^−1^	[39]
Sulfur-doped graphene/gold nanoparticles/screen-printed electrode	Linear sweep voltammetry	35.24–18,123 (ng mL^−1^)	15.39 ng mL^−1^	[40]
Ni-SPE	CV	0.25−25.0 μM	74.0 nM	[41]
Modified interdigitated gear-shaped working electrode design (LIG/G-PANI)	CV	0.1–100 μg/mL	0.0813 μg/mL	Current research
Modified interdigitated gear-shaped working electrode design (LIG/G-PANI)	EIS	0.1–100 μg/mL	0.0577 μg/mL	Current research
Modified interdigitated gear-shaped working electrode design (LIG/G-PANI)	Chrono-amperometry	0.25–100 μg/mL	0.105 μg/mL	Current research

### 3.6. Cortisol Detection in Human Serum

In this study, artificial human serum was used to assess the performance of the biosensor for cortisol detection under realistic biological conditions. Instead of phosphate-buffered saline (PBS), heat-inactivated human serum served as the medium for cortisol dilution. Prior to use, the serum was thawed, and a cortisol stock solution was added. Serial dilution was performed to prepare a range of cortisol concentrations, which were tested from low to high concentrations using CV.

**Figure 5:**
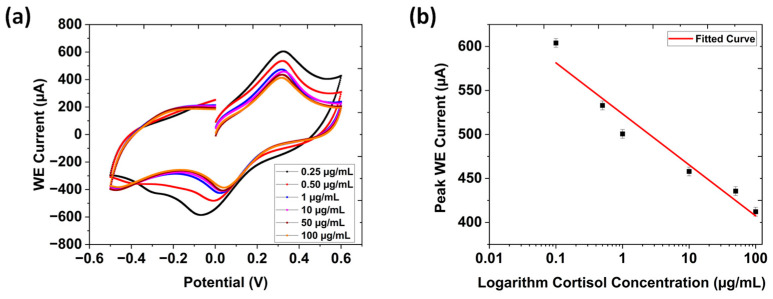
(**a**) CV responses obtained for interdigitated gear-shaped LIG/G-PANI sensor as a function of different cortisol concentrations (100 ng/mL to 100 μg/mL) in human serum. (**b**) Linear plot of the oxidation peak currents vs. different cortisol concentrations.

The electrochemical response in human serum followed a trend similar to that observed in PBS-based measurements, with a gradual decrease in current as cortisol concentration increased, as shown in Figure 5a. However, the overall current values in human serum were lower than in PBS. Moreover, when the concentration difference is less (i.e., 1 and 10 µg/mL), the current difference is not so significant. This difference is likely due to the higher viscosity and complex nature of the serum, which can hinder mass transport and slow down electron transfer at the electrode’s surface. The reduced current response in human serum compared to PBS is likely due to its higher viscosity and complex composition, which can hinder mass transport and slow down electron transfer at the electrode’s surface. This complexity may also limit the availability of cortisol at the electrode’s surface, affecting sensor performance [42]. The calibration curve of peak WE current versus the logarithm of cortisol concentration, exhibited in Figure 5b, indicates a regression equation of ΔI = −57.99 log [C] + 523.39 with strong linearity (R^2^ = 0.9486). Despite this, the sensor successfully detected cortisol across concentrations, demonstrating its applicability in complex biological samples.

### 3.7. Repeatability, Reproducibility, and Selectivity

Repeatability and reproducibility are critical parameters in evaluating the precision and reliability of a biosensor. They are typically quantified using the relative standard deviation (RSD%) or coefficient of variation (CV%) of the values obtained, ensuring minimal variance between successive measurements of the same sample [43].

**Figure 6:**
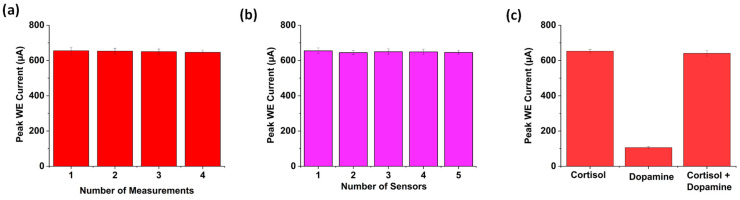
(**a**) Histogram showing the peak WE current variation with respect to the number of experiments. (**b**) Histogram showing the peak WE current variation with respect to different sensors for 0.50 µg/mL of cortisol in 0.1 M PBS (pH 7.4) containing ferri/ferrocyanide redox couplings. (**c**) Selectivity of the sensor developed for cortisol detection.

CV was performed four times under the same conditions, after applying 0.50 µg/mL of cortisol in 0.1 M PBS (pH 7.4) containing ferri/ferrocyanide redox couplings on the same electrode’s surface, in order to assess repeatability. The histograms for repeatability shown in Figure 6a indicate that the peak currents remained nearly identical across all four measurements, and the relative standard deviation (RSD) was calculated as 3.8%, indicating strong repeatability. For reproducibility, five separately prepared immunosensors from the same fabrication batch were examined using 0.50 µg/mL cortisol (Figure 6b). The current responses varied slightly, ranging between 645 and 655 µA, with an RSD of 5.85%, confirming good reproducibility across multiple sensor platforms. The manual preparation of the sensor can be the cause of the reproduced current’s smallest fluctuations.

To assess the selectivity of the developed biosensor, a ternary mixture containing 1 μg/mL cortisol and 0.5 mM dopamine was prepared. When tested in a cortisol-only solution using CV, the sensor exhibited a distinct current response. In contrast, in a dopamine-only solution, the biosensor produced only noisy data without a detectable oxidation peak. This can be attributed to the sensor’s immobilization with cortisol antibodies, which would have prevented any significant interaction with dopamine. However, the CV response from the ternary mixture showed a CV response almost similar to that found using a cortisol-only solution. This proves that the presence of interfering substances did not cause noticeable changes in current, confirming the high selectivity of the biosensor. The histograms of the peak current for the cortisol-only, dopamine-only and ternary mixtures are shown in Figure 6c.

## 4. Conclusions

In summary, we successfully developed a sensitive and selective electrochemical cortisol biosensor fabricated on Kapton film using a one-step direct laser-writing method. The incorporation of a graphene–polyaniline (PANI) ink layer significantly enhanced the sensor’s performance by increasing the active surface area, promoting efficient electron transfer, and enabling efficient current modulation in response to cortisol binding. Physical characterization via SEM and FTIR confirmed the successful integration of PANI onto the graphene surface. The antibody-based sensor demonstrated reliable detection of cortisol within a dynamic range of 0.1–100 µg/mL, with a low detection limit of 0.0577 µg/mL. Electrochemical measurements—including CV, DPV, and chronoamperometry—showed a decrease in current responses with rising cortisol concentrations due to the formation of an insulating immune complex. The EIS graph of charge-transfer resistance vs. logarithmic cortisol concentration indicates the increase in impedance, validating the previous result of the decrease in current. The sensor exhibited good repeatability and reproducibility, with relative standard deviation (RSD) values of 3.8% and 5.85% in PBS, respectively. The sensor also showed promise in detecting cortisol in artificial human serum. To extend this work in the future, we will focus on developing a wearable sensor and evaluate the sensor using human saliva, sweat, or serum, in addition to integrating it with a portable potentiostat for real-time, point-of-care applications. This work highlights the potential of electrochemical biosensing in monitoring cortisol levels and supporting the management of stress-related health conditions.

## Figures and Tables

**Table 1:** Values of different elements of the Nyquist plot for different concentrations of cortisol.

Cortisol Concentration	Solution Resistance (Rs)	Charge-Transfer Resistance (R_ct_)	Constant Phase Element (CPE)
0.25 µg/mL	13.2 Ω	126.41 Ω	Y_0_ = 2.33 n℧s^N^ N = 1.29
0.50 µg/mL	17.2 Ω	129.60 Ω	Y_0_ = 2.63 n℧s^N^ N = 1.34
1 µg/mL	17.5 Ω	130.79 Ω	Y_0_ = 2.64 n℧s^N^ N = 1.33
10 µg/mL	18.6 Ω	138.85 Ω	Y_0_ = 2.86 n℧s^N^ N = 1.36
100 µg/mL	31.8 Ω	155.30 Ω	Y_0_ = 1.56 n℧s^N^ N = 1.25

## Data Availability

The original contributions presented in the study are included in the article; further inquiries can be directed to the corresponding author.
